# PP01 Early Health Technology Assessments Of Health And Well-Being Returns On Investment In The Biobanks

**DOI:** 10.1017/S0266462324001752

**Published:** 2025-01-07

**Authors:** Clara Marquina, Melanie Lloyd, Wayne Ng, Jonas Hess, Sue Evans, Zanfina Ademi

## Abstract

**Introduction:**

Human tissue biobanks provide vital infrastructure to support both basic science and clinical research, but their economic value in terms of attributable population health gains is unclear. We evaluated the population health returns from investment in the Victorian Cancer Biobank (VCB). The VCB comprises five hospital-integrated sample repositories and a central lead agency located in Melbourne, Australia.

**Methods:**

This evaluation assigned monetary values to the health gains attributable to VCB-supported public-funded research. These were then compared to the total investment in VCB infrastructure since inception (2006 to 2022) to determine the return on investment (ROI). A time lag of 40 years was incorporated, recognizing the delay from investment to impact in scientific research. Health gains were therefore measured for the years 2046 to 2066, with a three percent discount rate applied. Health gains were measured in terms of disability-adjusted life years (DALYs) attributable to VCB-associated research, with monetary cost assigned via the standardized value of a statistical life year (AUD227,000 [USD149,883]).

**Results:**

The age-standardized DALY rate attributable to cancer was modeled for two standpoints: (i) extrapolating the current decreasing trajectory and (ii) assuming nil future improvement from current rates, with 33 percent of the difference attributed to scientific innovation. The proportion of the aggregate health gain attributable to VCB-supported research was estimated from the number of VCB-credited scientific publications as a proportion of total oncology publications over the same period. The AUD32,628,016 [USD21,554,571] of public funding invested in VCB activities over the years 2006 to 2022 generated AUD84,561,373 [USD55,868,539] total savings. Return on investment was AUD1.59 [USD1.05] for each AUD1 [USD0.66] invested.

**Conclusions:**

The VCB offers a strong return on investment in terms of population health impacts, justifying the use of public funds and supporting the use of biobanks to advance scientific research. Future health technology assessments could capture the total impact of research on the role of the biobanks attributed to research outputs.

